# Comparative Study of Various Immune Parameters in Three Bivalve Species during a Natural Bloom of *Dinophysis acuminata* in Santa Catarina Island, Brazil

**DOI:** 10.3390/toxins2051166

**Published:** 2010-05-25

**Authors:** Danielle Ferraz Mello, Luis Antonio de Oliveira Proença, Margherita Anna Barracco

**Affiliations:** 1 Laboratório de Imunologia Aplicada à Aquicultura, Departamento de Biologia Celular Embriologia e Genética, Universidade Federal de Santa Catarina, Florianópolis, Santa Catarina, Brazil; Email: danifmello@gmail.com; 2 Centro de Ciências Tecnológicas da Terra e do Mar, Universidade do Vale do Itajaí, Itajaí, Santa Catarina, Brazil; Email: luis.proenca@univali.br

**Keywords:** harmful algal bloom, Dinophysis acuminata, bivalves, immune parameters, Crassostrea gigas, Perna perna, Anomalocardia brasiliana

## Abstract

This study aimed to verify if *Dinophysis acuminata *natural blooms affected the immune system of three bivalves: the oyster, *Crassostrea gigas*, the mussel, *Perna perna*, and the clam, *Anomalocardia brasiliana*. Animals were obtained from a renowned mariculture farm in the southern bay of Santa Catarina Island during, and 30 days after (controls), an algal bloom. Various immunological parameters were assessed in the hemolymph of the animals: total and differential hemocyte counts, percentage of apoptotic hemocytes, protein concentration, hemagglutinating titer and phenoloxidase activity. The results showed that the mussel was the most affected species, with several altered immune parameters, whereas the immunological profile of clams and oysters was partially and completely unaffected, respectively.

## 1. Introduction

Harmful algal blooms (HABs) are a worldwide phenomenon caused by a variety of microalgal species, particularly dinoflagellates, which produce different kinds of biotoxins that can cause harm to humans and marine ecosystems [1]. In the last few decades, the occurrence of HABs has been increasing in frequency, intensity and geographical distribution due to both natural causes and human activities [2]. 

HABs are known to have important negative effects not only on marine environments but also on the economies of countries that participate in extracting and cultivating bivalves and fishes. Bivalves are filter-feeding animals that can bio-accumulate toxic compounds in their tissues. Therefore, the consumption of contaminated bivalves can represent a serious risk to their predators, including humans [1,3].

Due to the drastic impact that HABs represent to bivalve consumers, several monitoring programs of coastal areas have been implemented to detect algal blooms as early as possible to prevent the commercialization and consumption of contaminated bivalves. These programs have important positive effects on the mariculture sector of several producing countries, including Brazil, because they allow the country to develop without exposing the consumers to contaminated bivalves [4]. 

The coast of Santa Catarina in southern Brazil is the major Brazilian bivalve producer, with a production of about 13,000 tons/year of mussels (*Perna perna*) and oysters (*Crassostrea gigas*). This activity has a strong influence on the economy of the Santa Catarina region, as well as an important local social impact, as it represents a significant source of income to several small marine farmers who depend on this activity for their livelihood. 

Although large rates of mortality are not usually observed in cultivated bivalves during HAB outbreaks, the true effects of algal toxins on fish, shellfish and other marine organisms are still very poorly understood [1]. In contrast, the effects of HABs on human health have been widely studied, as has the mode of action of the different toxins. It is known, for example, that the ingestion of bivalves contaminated by the dinoflagellate, *Dinophysis acuminata*, causes health problems due to algal diarrheic shellfish toxins (DSTs) and promotes diarrhoea, nausea, vomiting and abdominal pain [5]. These symptoms are mainly caused by toxins that have accumulated in bivalve tissues, such as okadaic acid (AO) and other analogs, including dinophysistoxins, which are produced by some species of dinoflagellates from the *Prorocentrum* and *Dinophysis* genera.

Recent studies have suggested that bivalves, and particularly the cells of the bivalve immune system, can be adversely affected by HABs. In some cases, there was a clear modulation of hemato-immunological parameters, especially the cellular immune response [6,7,8,9,10,11,12,13,14,15,16,17,18]. However, most of these studies were carried out under controlled laboratory conditions, where animals are experimentally exposed to harmful algae, and very few studies were performed during natural HAB outbreaks [7].

Bivalve molluscs possess an innate immune system that is intimately connected to their blood, or hemolymph. The blood consists of circulating cells, called hemocytes, which are involved in cellular immune responses, and a liquid fraction, or plasma, which contains a variety of humoral factors related to humoral immune responses. Cellular and humoral hemolymph fractions work together to protect bivalves against infections and ensure their homeostasis [19]. 

Natural blooms of the dinoflagellate, *D. acuminata*, are increasing in an alarming frequency on the coast of Santa Catarina. These algal blooms are causing significant outbreaks in mariculture farms and consequently, are negatively affecting the economy of this region, which is the major bivalve producer in Brazil [4,20]. The aim of this study was to evaluate the effects of a natural bloom of *D. acuminata* on various hemato-immunological parameters of three bivalve species: the mussel, *Perna perna*, the Japanese oyster, *Crassostrea gigas, *and the clam, *Anomalocardia brasiliana.*

## 2. Materials and Methods

### 2.1. Animals and experimental design

Three adult bivalve species were used in this study: the brown mussel, *Perna perna* (shell height 85-95 mm), the Japanese oyster, *Crassostrea gigas* (90-100 mm), and the tropical clam, *Anomalocardia brasiliana* (25-35 mm). All animals (kept in lantern nets) were obtained from a renowned mariculture farm located in the southern bay of Santa Catarina Island in southern Brazil (27°38’56”S and 48°32’31”W) during a natural bloom of *Dinophysis acuminata* in April 2008, which lasted about 10-12 days. The animals were collected at the peak of the algal bloom (17,600 cells/L). Water samples were also collected at the marine farm and treated with lugol solution (1%) to determine the number of *D. acuminata *cells. Bivalves were transferred to the Laboratory of Immunology Applied to Aquaculture (LIAA), where the digestive glands were removed and hemolymph collected. Blood was withdrawn from the adductor muscle of 15 oysters, 15 mussels and 30 clams and pooled (3 pools from 5 animals each for *C. gigas* and *P. perna*, and 3 pools from 10 animals for *A. brasiliana*). One month after the algal bloom, water samples and animals (same numbers) were collected at the same location and used in the same analyses (reference animals).

**Figure 1:**
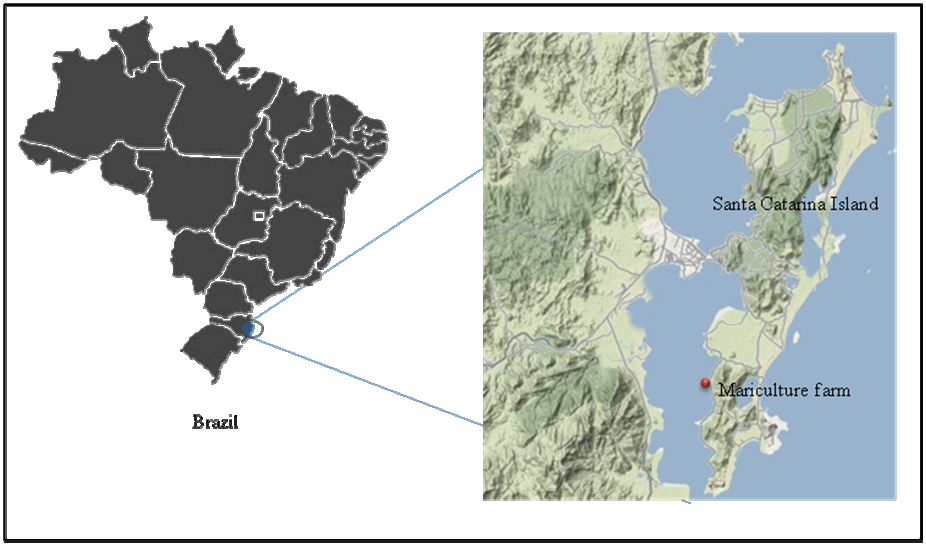
Mariculture farm in the southern bay of Santa Catarina Island where all bivalves were obtained.

### 2.2. Algal cell counts and determination of okadaic acid (OA) concentration in bivalve tissues

The number of *D. acuminata* cells was quantified after algal sedimentation using an inverted microscope according to the protocol described by Utermohl [21]. OA concentration was measured in the digestive gland extracts of mussels and oysters through liquid chromatography with mass spectroscopy (LC MS/MS). Tissue extracts were prepared by washing the digestive gland homogenates (2-3 g) with 20 mL of methanol (twice). Tissue extracts were then centrifuged at 3,000 × g for 10 min and filtered through 0.2 µm nylon filters. The chromatography was carried out on the Agilent LC system equipped with a fast LC Zorbax Eclipse XDB-C18 column. Okadaic acid was detected using an Applied Biosystem API 3200 Trap MS/MS detector calibrated with pure standards from NRC Canada, following settings obtained from Villar-Gonzáles *et al*. (2008). The OA concentration was then estimated for each whole bivalve body by applying a factor of 10.

### 2.3. Hemolymph preparation

The different hemolymph pools were divided into two parts: one part was fixed with 4% formaldehyde-MAS (modified Alsever solution: 27 mM sodium citrate, 336 mM NaCl, 115 mM glucose, 9 mM EDTA, pH 7.0) and was used to determine the hemograms and the percentage of apoptotic hemocytes; the other part was used to prepare total hemolymph (TH). TH was obtained by lysing the hemocytes through sonication (3 cycles of 7 s each, at 22.5 kHz/50 W, at 4 °C). The disrupted cell suspension was centrifuged (8,000× g for 30 min at 4 °C) and the supernatant or TH (exocytosed cell products + plasma) was removed and stored at −20 °C until use.

### 2.4. Hemograms-Total (THC) and differential (DHC) hemocyte counts

Total hemocyte counts (THC) were determined from fixed hemolymph pools through a Neubauer chamber. The relative percentage of the different hemocyte populations (DHC) was estimated by analyzing 200 cells of each fixed blood sample through a phase-contrast microscope.

### 2.5. Quantification of apoptotic hemocytes (AH)

The percentage of potentially apoptotic hemocytes was determined by using Hoechst 33258 staining (SIGMA). Fixed hemocyte monolayers were immersed in McIlvane buffer (0.1 M citric acid, 0.4 M disodium hydrogen phosphate, pH 5.5) for 5 min and then treated with a Hoechst solution in McIlvane buffer (1:1500) for 5 min. The slides were then mounted with coverslips and observed under a fluorescence microscope (365 nm). The percentage of apoptotic cells was estimated by examining 200 cells per sample and counting morphologically altered nuclei characteristic of apoptotic cells.

### 2.6. Hemagglutinating activity of total hemolymph (HA)

Samples of 50 μL of TH from the different pools were diluted serially in TBS-1 (50 mM Tris, 150 mM NaCl, 10 mM CaCl_2_, 5 mM MgCl_2_, pH 7.4) in 96-well microplates (U-shaped bottom) and incubated with the same volume of a 2% suspension of dog erythrocytes (in TBS-1) for 2 h at 20 °C in a humidified chamber. For controls, TH was replaced by TBS-1. The agglutinating titer (AT) was expressed as the reciprocal of the highest TH dilution showing positive agglutination. The titers were converted to log_2_ and the assays were performed in duplicate.

### 2.7. Determination of phenoloxidase (PO) activity

PO activity was determined spectrophotometrically through the formation of a red pigment (DOPA-chrome) by oxidation of the enzyme substrate, L-dihydroxyphenylalanine (L-DOPA). TH samples (50 μL) were diluted (v/v) in TBS-2 (50 mM Tris, 400 mM NaCl) and incubated with 50 μL of L-DOPA (3 mg/mL) in 96-well plates (flat bottom) at 20 °C. The reaction was carried out at pH 9.0, as alkaline pH is the most optimal enzyme inducer. The formation of DOPA-chrome was determined through a microplate reader (A_490_) after 5, 10, 20, 40 and 60 min. For controls, TH was replaced by TBS-2. One enzyme unit (1 U) corresponded to an increase of 0.001 in the absorbance per min, per mg of protein at 20 °C [22]. All assays were carried out in triplicate. 

### 2.8. Total protein concentration (PC)

PC was determined in the different TH pools according to the method of Bradford (1976), using bovine serum albumin (BSA) as a standard. The assays were carried out in triplicate.

### 2.9. Statistical analysis

The obtained results were first subjected to Bartlett's test to evaluate homogeneity of variances. The results of each immunoparameter were then compared by one-way ANOVA followed by Tukey’s post-test (mean comparison). For DHC and AH (percentages), the data were arcsin transformed. The results were considered significant at p < 0.05. Statistical analysis was performed using GraphPad Prism® software, version 5.0.

## 3. Results

### 3.1. Algal cell counts and okadaic acid (OA) concentration in bivalve tissues

No mortality was observed in any bivalve species during the natural bloom of *D. acuminata*. The number of *D. acuminata* cells dropped from 17,600 cells/L during the algal bloom (April 2008) to 0 cells/L one month after the bloom.

The concentration of OA was quantified only in mussel and oyster tissues. The concentration of OA in *P. perna* dropped from 87.9 µg/kg during the algal bloom to 1.7 µg/kg one month after it. On the other hand, the concentration of OA in the oyster tissues was about 12-times lower (6.8 µg/kg) than in mussels during the algal bloom. In agreement with these results, mussel tissue homogenates obtained during the *D. acuminata* bloom were positive in mouse bioassays. These results confirmed the high toxin accumulation in mussel tissues in contrast to the oysters. The tissue homogenates of *A. brasiliana* were negative in mouse bioassays*. *

### 3.2. Hemograms: THC and DHC

**Figure 2:**
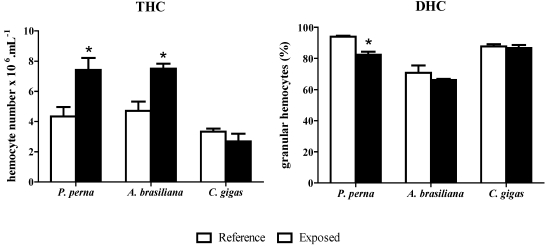
Total (THC) and differential (DHC) hemocyte counts in mussels (3 hemolymph pools from 5 animals), clams (3 pools from 10 animals) and oysters (3 pools from 5 animals) during a natural bloom of *Dinophysis acuminata* (black bars) and one month after the bloom (reference animals-white bars). Vertical lines represent standard errors and asterisks represent significant differences (p < 0.05) between groups from the same species.

During the natural algal bloom, the total hemocyte numbers (THC) of *P. perna* and *A. brasiliana* were significantly higher (70% and 60%, respectively) than in reference animals (Figure 2), but not in *C. gigas*, where THC did not vary. 

The three bivalve species contained the two characteristic hemocyte populations described in bivalves: hyaline hemocytes (HHs) and granular hemocytes (GHs). GHs were the predominant cell type in all species. *P. perna* was the only bivalve that had a significantly altered DHC during the *D. acuminata* bloom (Figure 2). The percentage of GHs dropped 12% in exposed mussels compared to reference mussels. On the other hand, the percentage of GHs in *C. gigas* and *A. brasiliana* remained unaltered from reference animals, and *A. brasiliana* exhibited the lowest overall values. 

### 3.3. Percentage of apoptotic hemocytes (AH)

**Figure 3:**
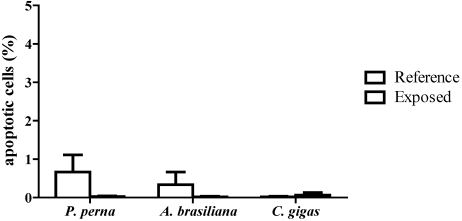
Percentage of characteristic apoptotic hemocytes (AH) in mussels (3 hemolymph pools from 5 animals), clams (3 pools from 10 animals) and oysters (3 pools from 5 animals) during a natural bloom of *D. acuminata* (black bars), and one month after the bloom (reference animals - white bars). Vertical lines represent standard errors.

Apoptotic hemocytes were determined by counting the altered nuclei characteristic of apoptotic cells visualized by Hoechst staining. All three bivalve species displayed a very low percentage (<1.5%) of apoptotic hemocytes during the *D. acuminata* bloom (Figure 3), and no significant differences in apoptosis were noted among the three bivalve species during the algal bloom. 

### 3.4. Hemagglutinating activity (HA)

**Figure 4:**
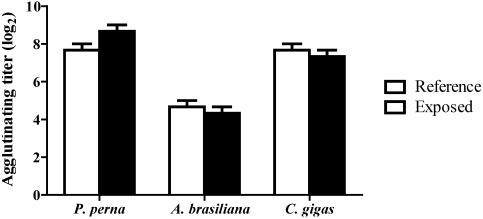
Hemagglutinating activity (HA) of the total hemolymph from mussels (3 hemolymph pools from 5 animals), clams (3 pools from 10 animals) and oysters (3 pools from 5 animals) during a natural bloom of *D. acuminata* (black bars) and one month after the bloom (reference animals-white bars). Vertical lines represent standard errors.

The hemagglutinating titer of TH against dog erythrocytes was different among the three bivalve species (Figure 4). However, the HA did not significantly vary within any bivalve species during or following the *D. acuminata* bloom (Figure 4). In *P. perna*, there was a trend to more elevated HA values in animals during the algal bloom.

### 3.5. Phenoloxidase (PO) activity

**Figure 5:**
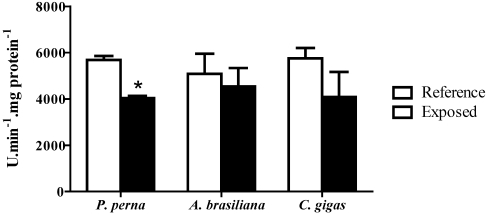
Phenoloxidase activity (PO) in the hemolymph of mussels (3 hemolymph pools from 5 animals), clams (3 pools from 10 animals) and oysters (3 pools from 5 animals) during a natural bloom of *D. acuminata* (black bars) and one month after the bloom (reference animals-white bars). Vertical lines represent standard errors and asterisks represent significant differences (p < 0.05) between groups from the same species.

PO activity decreased significantly (30%) in *P. perna* hemolymph during the *D. acuminata *bloom, but not in the other bivalve species. In oysters and clams, there was a trend to lower values compared to their respective reference animals (Figure 5).

### 3.6. Total protein concentration in hemolymph (PC)

**Figure 6:**
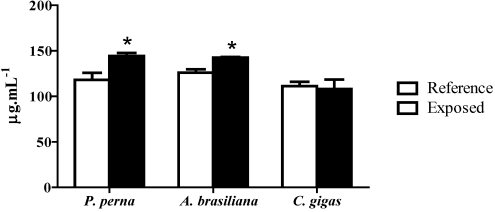
Protein concentration (PC) in total hemolymph of mussels (3 hemolymph pools from 5 animals), clams (3 pools from 10 animals) and oysters (3 pools from 5 animals) during a natural bloom of *D. acuminata* (black bars) and one month after the bloom (reference animals-white bars). Vertical lines represent standard errors and asterisks represent significant differences (p < 0.05) between groups from each species.

The PC in the hemolymph from *P. perna *and *A. brasiliana* increased significantly (22% and 13%, respectively) during the bloom of *D. acuminata*, whereas in the oyster it remained unchanged when compared to reference animals (Figure 6).

## 4. Discussion

As previously discussed, harmful algal blooms (HABs) have become more and more prevalent on the coast of Santa Catarina, which is the major producer of bivalves in Brazil. In recent years, blooms of the dinoflagellate, *D. acuminata*,have been particularly rampant [20] and are threatening the mariculture activity in this locality. In this study, we evaluated whether blooms of *D. acuminata*, which produce the okadaic acid (OA) toxin, could cause immunosuppression in bivalves, making them more vulnerable to pathogens and/or other stressful agents in the environment. Very few studies have addressed the effects of HABs and/or their toxins on the immune system of bivalves. The results of these studies suggest that bivalves can in fact be affected during HABs, and several abnormalities can be observed in the immune responses of bivalves [6,7,8,9,10,11,12,13,14,15,16,17,18].

Interestingly, the three bivalve species selected in this study responded in a different manner during the bloom of *D. acuminata*,even though they were collected at the same time and in the same place. These results suggested that each bivalve species had different rates of accumulation and/or clearance of the *D. acuminata *toxin, okadaic acid (OA). *P. perna* was the species that showed the greatest capacity to accumulate OA and/or the lowest ability to clear this toxin. Similar results were observed on the Santa Catarina coast during another *D. acuminata *bloom in 2007; here mussels accumulated 10-times more toxin than *C. gigas* [4]. Other species of mussels, such as *Mytilus galloprovincialis* from the Adriatic Sea, seem also to accumulate much more DSTs (diarrheic shellfish toxins: dinophysistoxin and OA) in their tissues, as revealed by bioassays, than clams (*Chamelea gallina*, *Tapes decussata *and *Venus verrucosa*) [5]. Furthermore, *M. galloprovincialis* from Mutso Bay in Japan had the capacity to accumulate much higher levels of OA in their tissues than the scallop, *Patinopecten yessoensis* [23]. Sidari *et al.* [24] suggested that *M. galloprovincialis* has a feeding preference for *Dinophysis* dinoflagellates and that they can easily digest these algae. Very recently, Lindegarth *et al.* [25] reported that another mussel, *Mytilus edulis*,could also accumulate DSTs at a much higher concentration (10- to 50-times more) and much faster than the oyster, *Ostrea edulis*, suggesting that the digestive tracts of both species have different rates of toxin metabolism. On the other hand, both species have a similar rate of clearance of these toxins.

Concerning the immune system of the three bivalve species, the most pronounced results were seen in the mussel, *P. perna*, which accumulated the highest concentration of toxins in its tissues during the *D. acuminata* bloom. Several hemato-immunological parameters were altered in the mussel, whereas no changes occurred in the oyster, *C. gigas*, and the clam, *A. brasiliana*, showed an intermediate phenotype. Unfortunately, we have no data on the accumulation of OA in the tissue of clams, but they were negative in bioassays. However, the limit of toxin detection is very high in bioassays, and so we presume that clams did accumulate OA in sufficient levels to cause some alteration in their immune profile but not enough to give positive results in bioassays. These results could be due to different rates of *D. acuminata* filtration among the three bivalve species and/or different rates of OA metabolism. 

A number of hemato-immunological parameters were evaluated in this study. The total cell count (THC) is one of the most widely used parameters to assess bivalve health status because the number and proportion (DHC) of circulating hemocytes have been shown to change under stressful conditions [26]. In effect, there was a marked increase in the THC of *P. perna* (70%) and *A. brasiliana* (60%), but not *C. gigas* during the *D. acuminata* bloom. In contrast to *P. perna*, the THC of the other mussel, *M. edulis*, dropped when exposed to the dinoflagellate, *Alexandrium fundyense*, which produces PSTs (paralytic shellfish toxins) [12], but did not change in the presence of the dinoflagellates, *Prorocentrum minimum* and *Karlodinium veneficum* [11,14]. A similar situation was observed in the clam, *Ruditapes philippinarum*, where the THC decreased upon exposure to the dinoflagellate, *P. minimum* [13], but did not change in the presence of the algae, *Karenia selliformis* and *K. mikimotoo*, which produce NSTs (neurotoxic shellfish toxins) [8,10]. In oysters, the THC could also be modulated upon exposure to different HABs. Both the THC and percentage of granular hemocytes in *C. virginica* increased significantly after experimental exposure to *P. minimum* [7] but did not vary in the presence of the dinoflagellates, *A. fundyense* and *A. catenella* [27]. These results suggest that the effect on the immune system and the modulation of immune parameters depend both on the species of harmful algae and the species of bivalve. 

Regarding DHC, a significant variation in the hemocyte populations was observed only in *P. perna* during the *D. acuminata* bloom*. *There was a decrease in the percentage of GHs (12%), the major immunocompetent cell type, which may suggest an immunosuppression of the mussels. Because the decrease in GHs was accompanied by an increase in THC, we can assume that there was an induction of the production and/or release of hemocytes from hematopoietic tissue during the natural bloom, with the preferential release of hyaline hemocytes (HHs). Alternatively, the two cell populations (HH and GH) could be released in equivalent numbers, but part of the GHs migrated to and infiltrated in tissues that were in contact with the algae/toxins, such as gills and intestine. Indeed, the preferential migration of GHs to tissues in contact with pathogens [28] or toxic algae [12] has been previously demonstrated in some bivalves.

Apoptosis or programmed cell death is an essential mechanism in the regulation of homeostasis and in immune defence in most organisms. Changes in the percentage of apoptotic hemocytes were already reported in bivalves during HABs [10,12]. In this study, we did not find any significant variation in the percentage of apoptotic hemocytes during the *D. acuminata* bloom in the three bivalve species. Similarly, no increase in apoptotic hemocytes was seen in *M. edulis* exposed to *P. minimum* [11] and *K. veneficum* [14]. 

The total protein concentration (PC) in the hemolymph has also been used to evaluate health status in bivalves, as hemocyte activation by pathogens or other stressors can cause the release of immune mediators into the plasma [29,30,31]. Consistent with this, we observed an increase in PC in the hemolymph of mussels and clams (22% and 13%, respectively) but not oysters during the algal bloom. In contrast, no alteration in PC concentration was found in the clam, *R. philippinarum*, upon exposure to *K. selliformis* and *P. minimum* [8,10,13]. Increases in PC concentration could also indicate a process of histolysis triggered by toxins from algae or other pollutants, as was observed in mussels from contaminated sites in the Mediterranean Sea [29].

Naturally occurring lectins in bivalve hemolymph are considered to be recognition proteins (PRPs) that recognize molecular patterns (sugars) on the surface of pathogens, and the concentration of these PRPs might be enhanced during infections or under stressful conditions [32]. In the present study, there were no significant differences in the hemagglutinating titers in any bivalve during the algal bloom. In the clam, *R. philippinarum*, the haemagglutinating activity also did not vary in the presence of different species of harmful algae [8,10,13].

The prophenoloxidase (proPO) system is being increasingly used to assess bivalve health status in response to pathogens or stressful conditions [33]. In the present study, there was a decrease in phenoloxidase (PO) activity only in *P. perna* hemolymph during the algal bloom. These results could be due to the decrease in the percentage of circulating GHs, as PO is produced and stored in GHs [34]. In contrast to our results, an increase in PO activity was reported in diploid and triploid *C. gigas* oysters exposed to the dinoflagellate, *Alexandrium minutum*, producing PSTs [35].

In conclusion, the results of this initial study showed that each selected bivalve species behaved differently in the presence of the *D. acuminata* natural bloom, but no animal mortality was recorded. The mussel, *P. perna*, was the species most affected immunologically and accumulated the highest concentration of okadaic acid in its tissues. The oyster, *C. gigas*, was the least affected species, exhibiting low tissue toxin concentration and little immunological alteration. Unfortunately, we could not measure OA concentration in the clam, *A. brasiliana. *However, we presume that these animals did effectively accumulate a certain amount of toxins because their immune profile was affected, although to a lesser extent than the mussels. Altogether, these results indicate that *D. acuminata *blooms can have effects on the immune system of bivalves, especially when they accumulate high concentrations of toxins in their tissues, and this capacity varies according to the bivalve species. It would be interesting to better establish which bivalves are immunologically more sensitive to which toxic algae during the periods of algal blooms in Santa Catarina; this could serve as a strategy to better control potential infections or other stressful conditions that may further affect cultivated bivalves already immunodepleted by algal blooms.
